# Supplementary material for: Regulation of Jacobaea vulgaris by varied cutting and restoration measures
Source: PLoS One. 2022 Oct 6;17(10):e0248094. doi: 10.1371/journal.pone.0248094 (PMC9536583; doi:10.1371/journal.pone.0248094)
Supplement: S1 Table — Field properties (name, location, region, applied mower). (DOCX) [file pone.0248094.s002.docx]

**Table S1.** Characteristics of the study sites

Field properties (name, location, region, applied mower) of the eight study sites, and the region in which they were located (coordinate system: ETRS_1989_UTM_Zone_32N; EPSG: 25832).

| Field name | X | Y | Region | mower |
| --- | --- | --- | --- | --- |
| Arpsdorf | 553935 | 5986520 | Moraine | Bar mower |
| Berkenthin | 610007 | 5954718 | Hill land | Bar mower |
| Kesdorf | 608728 | 5991168 | Hill land | Mulcher |
| Preetz | 581177 | 6009517 | Hill land | Mulcher |
| Neversdorf | 577021 | 5975037 | Hill land | Bar mower |
| Schafhaus | 586183 | 5967125 | Moraine | Bar mower |
| Westerwohld | 513654 | 6001401 | Moraine | Bar mower |
