# Supplementary material for: Regulation of Jacobaea vulgaris by varied cutting and restoration measures
Source: PLoS One. 2022 Oct 6;17(10):e0248094. doi: 10.1371/journal.pone.0248094 (PMC9536583; doi:10.1371/journal.pone.0248094)
Supplement: S2 Table — Herbs and grasses applied in moraine and hill land. (DOCX) [file pone.0248094.s003.docx]

**Table S2.** Applied seed mixture on *biodiversity* and *combination* treatments

| **Moraine** | **Hill land** |
| --- | --- |
| **herbs** | |
| *Achillea millefolium* | *Achillea millefolium* |
| *Centaurea jacea* | *Centaurea jacea* |
| *Daucus carota* | *Daucus carota* |
| *Dianthus deltoides* | *Dianthus deltoides* |
| *Galium album* | *Galium album* |
| *Hypericum perforatum* |  |
| *Knautia arvensis* |  |
| *Leucanthemum vulgare* | *Leucanthemum vulgare* |
| *Medicago lupulina* |  |
| *Plantago lanceolata* | *Plantago lanceolata* |
| *Ranunculus acris* | *Ranunculus acris* |
| *Silene dioica* | *Silene dioica* |
| *Silene latifolia* | *Silene flos-cuculi* |
| *Silene vulgaris* | *Silene vulgaris* |
| *Stellaria graminea* | *Stellaria graminea* |
| *Trifolium pratense* | *Trifolium pratense* |
| *Thymus pulegioides* | *Thymus pulegioides* |
|  |  |
| **grasses** | |
| *Agrostis capillaris* | *Agrostis capillaris* |
| *Anthoxanthum odoratum* | *Anthoxanthum odoratum* |
| *Arrhenatherum elatius* | *Arrhenatherum elatius* |
| *Bromus hordeaceus* | *Bromus hordeaceus* |
| *Briza media* | *Briza media* |
| *Festuca guestfalica (ovina)* |  |
| *Festuca nigrescens (rubra)* | *Festuca nigrescens (rubra)* |
| *Lolium perenne* | *Lolium perenne* |
| *Poa pratensis* | *Poa pratensis* |
