# Supplementary material for: Regulation of Jacobaea vulgaris by varied cutting and restoration measures
Source: PLoS One. 2022 Oct 6;17(10):e0248094. doi: 10.1371/journal.pone.0248094 (PMC9536583; doi:10.1371/journal.pone.0248094)
Supplement: S2 Fig — In the box plots, middle lines represent the median, boxes represent the first and third quartiles, lower and upper bars represent the minimum and the maximum and points represent outliers (i.e. points above 1.5 SD). No significant differences were found. (DOCX) [file pone.0248094.s005.docx]

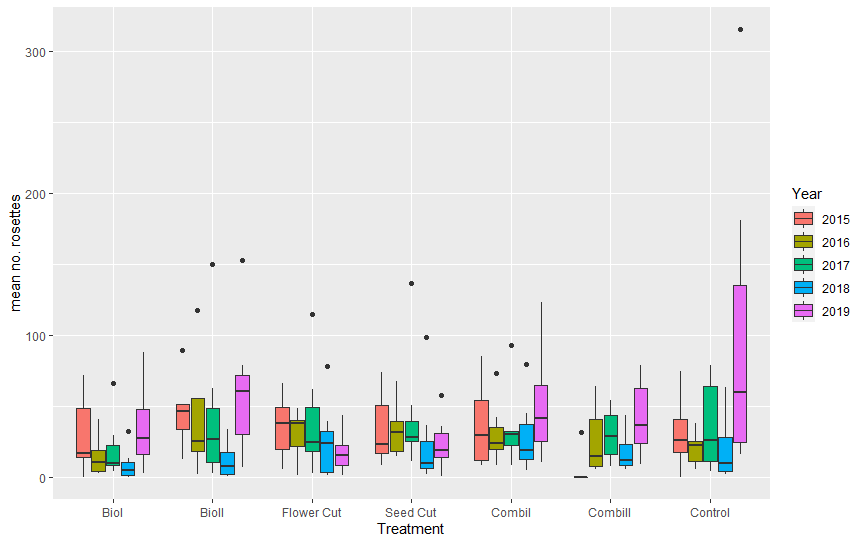


**Fig S2.** **Development of rosette numbers according to treatment and year.** In the box plots, middle lines represent the median, boxes represent the first and third quartiles, lower and upper bars represent the minimum and the maximum and points represent outliers (i.e. points above 1.5 SD). No significant differences were found.
