# Supplementary material for: Regulation of Jacobaea vulgaris by varied cutting and restoration measures
Source: PLoS One. 2022 Oct 6;17(10):e0248094. doi: 10.1371/journal.pone.0248094 (PMC9536583; doi:10.1371/journal.pone.0248094)
Supplement: S3 Fig — In the box plots, middle lines represent the median, boxes represent the first and third quartiles, lower and upper bars represent the minimum and the maximum and points represent outliers (i.e. points above 1.5 SD). Different letters indicate significant differences between the treatments (TukeyHSD, P ≤ 0.05). (DOCX) [file pone.0248094.s006.docx]

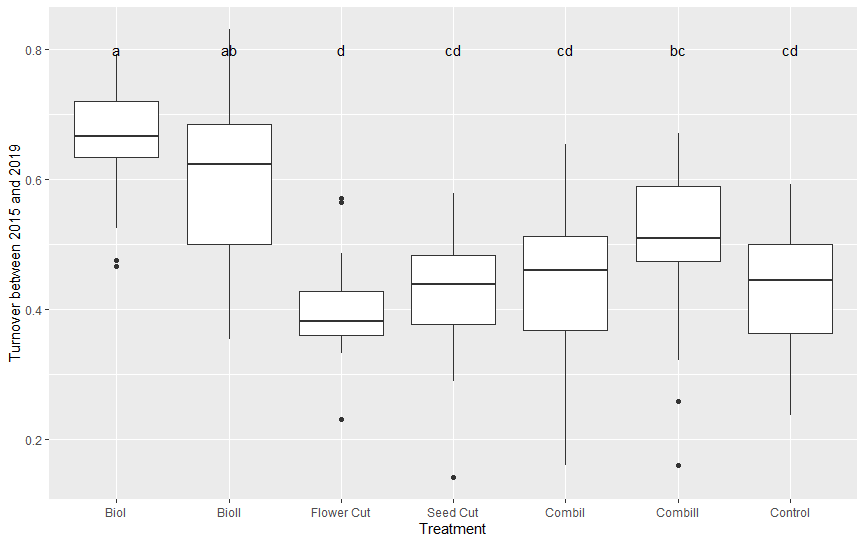
**Fig S3.** **Turnover rates for treatments.** In the box plots, middle lines represent the median, boxes represent the first and third quartiles, lower and upper bars represent the minimum and the maximum and points represent outliers (i.e. points above 1.5 SD). Different letters indicate significant differences between the treatments (TukeyHSD, P ≤ 0.05).
