# Supplementary material for: Regulation of Jacobaea vulgaris by varied cutting and restoration measures
Source: PLoS One. 2022 Oct 6;17(10):e0248094. doi: 10.1371/journal.pone.0248094 (PMC9536583; doi:10.1371/journal.pone.0248094)
Supplement: Attachment — Submitted filename: rebuttal_plosone_twdc_twd.docx [file pone.0248094.s009.docx]

Dear Editor and Reviewers,

We thank you very much for your valuable efforts and helpful comments and critiques. Based on both reviews we created an updated and improved manuscript.

Further down, we would like to directly address discussed points, with your critique stated repeated (following R1/R1) and our answer (following A). Minor points were track changed directly in the manuscript.

In any case, we thank you very much for taking our manuscript into consideration anew and thank you again for your helpful critique and comments.

Yours sincerely

Henrike Möhler

_______________________

Reviewer 1:
R1: Would you define "milling"? I'm not familiar with this practice in the context of soil disturbance.

A: We thought “milling” was defined as very shallow tillage with a rotary tiller. However, I guess that this was a wrong assumption. We replaced “milling” with “tilling”. And added the rotary tiller in the methodology part.

R1: Though you detected trends, the treatments didn't significantly affect J. vulgaris. I was wondering about site factors that may have interacted with management to dilute impacts. You control for "site" as a random factor in population growth (L195-196), but it's not clear that an interaction between site and treatment has been considered.

A: We agree with you that site factors diluted potential effects of management. We considered interaction between sites and treatment but did not find significant interaction and added that in the manuscript.

R1: Do you think site differences in pressures affecting recruitment and transitions among life stages may lead to different advantages of treatments among sites? (for instance, see Rand et al. 2020 Oecologia 193:143-153) Some of the concepts you reference around precipitation in the discussion (e.g., L410-412) would also be applicable to this question.

A: Yes, we do think that site-specific differences affect recruitment and transitions between life stages. Rand et al. 2020 had a vital point. Therefore, we added this reference and discussion point.

R1: Because it's wind dispersed, do you think proximity of blocks might muddy treatment effects on seedling recruitment? That is, were they sufficiently separated to minimize the invasion/seed dispersal from adjacent plots?

A: Definitely, we think that plots were still to close to each other. It is unrealistic that all upcoming seedlings in the two-cut-treatment originated from the soil. It is more probably that they arrived from nearby sites with different management. We added that point in the discussion.

R1: L41: Should this be overall plant diversity? As it includes some introduced seed, some ruderal weeds, etc.

A: Your right, we changed it accordingly.

R1: L133- each treatment was implemented over a 90 m2 area, so each site consisted of seven 90 m2 blocks?

A: It depends on what you define as a site. The pastures themselves varied in their sizes as they were not designed for the study but vice versa. Thus, the sites actually studied consisted of 7 90m² blocks but the sites themselves were much bigger.

R1: L148: How did you choose additional seed to mix?

A: We did not decide which seeds to add, but the funding body of our study decided that upon availability of authochthonus seeds it had produced in that year.

R1: L152: Seems out of place. Should this go up to 149-150?

A: We do not quite understand why and what exactly is not in place. As the sentences seem logical in their order, following the order of treatments established in the table 1, we refrain from changing the order.

R1: L179: I'm not sure where the pooling is occurring. I assume you mean that each site had a population growth rate in each treatment and year, and you're pooling your three plots within each block to each treatment?

A: Pooling occurred at the single stages. We assumed to have studied one huge population. Therefore, we added all seedlings from all sites and subplots and all rosettes and flowering plants. From that pooled data lambda was calculated. The error bars in the figure for mean lambda originate from boots trapping the data.

R1: 189 (analysis): may be helpful to organize around the two questions in the intro. I'm not sure exactly what is being tested at times (among years, treatments, at what level replication occurs). For instance, is site treated as a random factor in comparing the number of plants among treatments? (L201-205)
Confusion regarding ANOVAS and what's tested (e.g., L234-5)

A: Your correct, this part was not well described, we tried to reformulate our statistical approach. We used different approaches for the analysis of population growth and the analysis of numbers of individuals (a less elegant one in the latter case).

R1: L249 (population results): why not do mixed models with population growth? (site effects/interactions)

A: We did mixed models for population growth. In these models you have to decide which factors are random. Thus, we did check for interactions with factors that were defined as random. However, we did an ANOVA for all individuals regardless of stage and defining there every factor as equally important (year, treatment, site). When this ANOVA was carried out no significant interaction was found.

R1: L254-257: high then low growth rates: do you think this emerges from seedling flushes or from more flowering adults? Just curious.

A: We think that is due to seedling flushes as the number of flowering adults did not show that high ups and downs.

R1: L260-263: Not sure where this rainfall data and linear analysis comes from? Please add to supplement, or provide a reference.

A: Your right, we forgot to name the source of the data. It is from a German weather service called wetterkontor. We added the reference.

R1: L421: Do you think that even if rosette-rosette is not be critical to growth, it might maintain a potential pool through time that allows populations to persist even when not growing (L272-274)? Certainly the above suggests there's a holdover in flowering and expansion in droughty conditions.

A: Your right rosettes as well as flowering plants even if they do not shed seed are critically to the persistence of the population in all cutting treatments.

R1: L353: Does milling eliminate standing cover?

A: Yes, it does. But as tillage is not very deep roots or seeds within the soil can regenerate more easily than in classical deep tillage.

R1: L430: Soil disturbance doesn't seem as beneficial to site quality improvement, sowing and mowing (more frequently) does

A: I am not quite sure what you mean with site quality improvement and for which plant.

R1: L464: Why soil fertility? Can you add a couple of sentences here to provide context for this assertion?

A: We try to find explanations for why grass cover and occurrence of ruderals is more prevalent in the non-treated control plots. One factor that may be important is soil fertility that enables dominance of few generalistic plant species over other plants that might be suppressed by competition of the dominant plant species. Most of the study sites were located on ex-arable land, where fertilizers have been used in high quantities. Thus, soil fertility in all plots is higher than on sites, which were not used as fields before. This nutrient load might enable grasses and ruderal plants to thrive over the less competitive plants. However, if competition is changed via mowing, less competitive plants may take profit from that and are able to coexist with other plants.

Figures and Tables:
R1: Table 1: may be helpful to categorize treatments to highlight comparisons, e.g., columns: disturbance|frequency of disturbance|seeding|frequency of seeding| seed mix. Especially as hand sowing seemed to make a difference, but not drilling (L470)

A: We rearranged table 1 accordingly.

R1: L174: should this table have a legend? And should retrogression be included? L177 leaves out reference to "R", which is included in both the figure and table. (Figure 2: Is "R" retrogression?)

A: The legend to the table is part of the figure caption. Your right, that we forgot to mention “R”. We added that information.

R1: Figure 6: Bar colors for each category? (L277: bars are treatment and controls?)

A: Sorry that we forgot the colors. We added that information.

Check that figure categories are ordered consistently - Figures 3 and 8 are ordered differently.

A: Thanks for that advice! We changed figure 3 accordingly.

A few minor typos questions:
R1: L38 "Its control..."

Done

R1: L114: "vegetative" bud?

Done

R1: L126-7: "...very high with an (?) average....at the experiment's start"

Done

R1: L324: sowing in pulses? Or sowing pulses?

Done

R1: L338: no comma after milling

Done

R1: L385: orphaned "r" after "...causing..."

Done

R1: L399: ...compensated for this mortality

done

Reviewer #2

R2: describe methods in abstract and change conclusion

A: done accordingly

R2: L112: how long?

A: studies report from longevity up to 20 years

R2: Negative points: First, the work found no differences for the treatments tested, but built a conclusion as if it had found big differences. Therefore, reformulate the writing of the text based on the data found and do not make conclusions that the data do not support.

A: We down-toned treatment effects in the discussion. Nevertheless, we did find small significant effects and trends. Furthermore, significant differences within the biodiversity treatments are not only relatable to the years. As tillage as a management tool at first reduced the number of rosettes and flowering plants a lot for all years studied.

R2: Figures need better presentation, especially figures 5 and 6.

A: Sorry we are not sure what you are aiming at; we already changed the figures according to reviewer 1; an in case you can be more concrete we are happy to try to further improve them.

R2: Very long conclusion and nothing objective (it is not the time for discussion). It needs more objectivity.
Restructure the text according to the data found.

A: We restructured the text and put aspects from the conclusion in the discussion part. However, as conclusion goes hand in hand with an outlook, we refrain from eliminating the part with the insects as we think that this aspect has to be kept in mind for further studies.
