# Supplementary material for: Regulation of Jacobaea vulgaris by varied cutting and restoration measures
Source: PLoS One. 2022 Oct 6;17(10):e0248094. doi: 10.1371/journal.pone.0248094 (PMC9536583; doi:10.1371/journal.pone.0248094)
Supplement: Attachment — Submitted filename: Response to reviewersms3Henrike.docx [file pone.0248094.s010.docx]

Dear Editor and Reviewers,

We thank you very much for your time, efforts and helpful comments and critiques. Taking all of it into account we created an updated and improved manuscript.

We checked references anew and found no inconsistencies.

As proposed by reviewer 1 we replaced fertility with fecundity [L169-170 & L179-180, L395], corrected the numbering of questions in L201 and described methods we used for the calculation of "elasticity” and “evenness”, which was indeed an essential we forgot to mention in the first place. Thanks for the advice!

Furthermore, we had a second native English speaker check the manuscript and hope that it is in an understandable adequate language now.

We thank you for taking our manuscript into consideration anew and thank you again for your helpful critique and comments.

Yours sincerely

Henrike Wiggering, Tobias Donath, Tim Diekötter
