# Supplementary material for: Regulation of Jacobaea vulgaris by varied cutting and restoration measures
Source: PLoS One. 2022 Oct 6;17(10):e0248094. doi: 10.1371/journal.pone.0248094 (PMC9536583; doi:10.1371/journal.pone.0248094)
Supplement: Attachment — Submitted filename: Response to reviewersms4Henrike.docx [file pone.0248094.s011.docx]

Dear Editor and Reviewers,

We thank you very much for your helpful critiques! Taking your comments into account we created an updated and improved manuscript.

We added the minimum number of weeks in which the biodiversity treatments were fenced.

We added the lamda symbol in line 192.

We cited the Red List index used in the methods section (line 161, Mierwald 2006) and added the citation in line 312.

It is an interesting question how long seeds lie dormant. From our own experience there is no dormancy as we found new seedlings very soon after seed shedding. Moreover, we had a Bachelor Thesis treating the topic of the relevance of the seed bank and found that there were relatively little seeds in the seed bank but most seedlings resulted from the seed rain. Thus, we would expect that seed rain is more relevant than regeneration via seeds from the seed bank. However, it is very likely that both seedbank and wind-dispersal are contributing to new seedlings.

We completed the citation of Eisele in line 418.

We thank you for taking our manuscript into consideration anew and thank you again for your helpful critique and comments.

Yours sincerely

Henrike Wiggering, Tobias Donath, Tim Diekötter
